# A Brief Assessment on Recent Developments in Efficient Electrocatalytic Nitrogen Reduction with 2D Non-Metallic Nanomaterials

**DOI:** 10.3390/nano12193413

**Published:** 2022-09-29

**Authors:** Muhammad Shahid, Hafiz Muhammad Asif Javed, Muhammad Irfan Ahmad, Akbar Ali Qureshi, Muhammad Ijaz Khan, Maha Abdallah Alnuwaiser, Arslan Ahmed, Muhammad Azhar Khan, El Sayed Mohamed Tag-ElDin, Arslan Shahid, Aiman Rafique

**Affiliations:** 1 Nanomaterials and Solar Energy Research Laboratory, Department of Physics, University of Agriculture Faisalabad, Faisalabad 38000, Pakistan; 2 Department of Mechanical Engineering, Bahauddin Zakariya University, Multan 60000, Pakistan; 3 Department of Mechanics and Engineering Science, Peking University, Beijing 100871, China; 4 Department of Mechanical Engineering, Lebanese American University, Beirut P.O. Box 13-5053, Lebanon; 5 Department of Chemistry, College of Science, Princes Nourah Bin Abdulrahman University, Riyadh 11671, Saudi Arabia; 6 Department of Mechanical Engineering, COMSATS University Islamabad, Wah Campus, Rawalpindi 47010, Pakistan; 7 Department of Physics, The Islamia University of Bahawalpur, Bahawalpur 63100, Pakistan; 8 Faculty of Engineering and Technology, Future University in Egypt, New Cairo 11835, Egypt

**Keywords:** electrocatalytic conversion, nitrogen fixation, synthetic ammonia, nitrogen reduction

## Abstract

In recent years, the synthesis of ammonia (NH_3_) has been developed by electrocatalytic technology that is a potential way to effectively replace the Haber–Bosch process, which is an industrial synthesis of NH_3_. Industrial ammonia has caused a series of problems for the population and environment. In the face of sustainable green synthesis methods, the advantages of electrocatalytic nitrogen reduction for synthesis of NH_3_ in aqueous media have attracted a great amount of attention from researchers. This review summarizes the recent progress on the highly efficient electrocatalysts based on 2D non-metallic nanomaterial and provides a brief overview of the synthesis principle of electrocatalysis and the performance measurement indicators of electrocatalysts. Moreover, the current development of N_2_ reduction reaction (NRR) electrocatalyst is discussed and prospected.

## 1. Introduction

With the evolution of the world economy and rapid growth of the population, humankind is facing great challenges such as energy shortages, food crises, and environmental problems [1]. NH_3_ has great advantages as a clean energy source and can also act as a stable compound for hydrogen storage, transportation, and as an important part of fertilizer production [2]. NH_3_ is the raw material for the manufacture of synthetic dyes and polymers, as well as explosives in other fields such as military defense and construction [3,4]. At present, the world’s largest industrial ammonia synthesis technology was invented by the German scientists Haber and Bosch in 1909 and implemented in 1930 [5]. The Haber–Bosch approach has also been a huge source of ammonia in the world to date. However, with the increasing consumption of fossil energy and environmental problems brought about by the Haber–Bosch industrial method, there is an urgent obligation to introduce a sustainable and green ammonia synthesis approach. This is mainly because the highly pure hydrogen (H_2_) that is needed by the Haber–Bosch industry consumes vast amounts of fossil fuels (mainly natural gas), and industrial synthetic ammonia emits a large amount of greenhouse gases (CO_2_). Meanwhile, industrial synthesis technology consumes only 1–2% of the energy supplied by human power [6]. In addition, in Haber–Bosch industrial technology, nitrogen and hydrogen molecules are converted into NH_3_ in the presence of high temperature and pressure, in which pressure is approximately 20–40 MPa and temperature is 400–600 °C [7,8].

In recent years, the electrochemical nitrogen fixation, utilizing abundant sources of N_2_ and renewable electricity to convert N_2_ into high value-added products NH_3_ at the cathode, is a very promising method [9]. Under mild conditions, N_2_ can be effectively reduced in the water electrolyte by the electrocatalyst. Fortunately, nitrogen reduction reaction (NRR) has achieved a great success in practice. However, the high stability of N_2_ poses a great challenge for electrocatalytic reduction. The bond energy of nitrogen, which is approximately 945 kJ/mol of the N_2_ molecule, needs to be destroyed [10]. The competitive reaction in the electrolyte caused by hydrogen evolution reaction (HER) thereby limits the current effect of electrocatalytic N_2_ reduction and the low selectivity [11]. Therefore, the limited ammonia was synthesized by N_2_ and H_2_O due to low nitrogen reduction reaction and weak catalytic activity.

Recently, two-dimensional nanomaterials have attracted considerable attention in the fields of light, electricity, catalysis, energy storage, and sensors due to their special chemical and physical characteristics [12,13,14]. As is well known, a 2D material has a structure that is not limited in the lateral direction and is as thin as one or a few atoms [15]. The 2D materials form an ordered network structure through in-plane interaction and weak bonding forces. The development of this type of material was achieved by many researchers after Novoselov, Geim, and colleagues won the Nobel Prize in 2004 [16]. Novoselov et al. synthesized and reported graphene for the first time, and layered graphene was the beginning of the rise and development of 2D materials. In various applications of 2D nanomaterials, some 2D nanomaterials and their compounds have been widely used in the field of electrocatalysis [17]. Additionally, on the basis of the advantages of 2D materials, many excellent electrocatalysts have been developed, which are used in CO_2_ reduction [18], HER [19], etc., and have recently proved their superiority as electrocatalysts for an extensive range of reactions, especially for H_2_O and N_2_ as reactants. The emerging catalysis reaction is the synthesis of ammonia by electrocatalyst. The excellent progress in the past decade has made 2D nanomaterials-based electrocatalysts the most important type of nanostructured electrocatalysts [17,20]. Two-dimensional non-metallic nanomaterials have attracted considerable interest in a series of electrochemical reactions due to their economic feasibility, environmental friendliness, non-corrosiveness, and unique physical and chemical properties. At present, there are many types of NRR electrocatalysts based on 2D nanomaterials, such as precious-metal-based nanomaterials [21], transition-metal-based nanomaterials [22], and 2D-based non-metallic nanomaterials which have received special attention [23]. Typical examples are graphene and its functional derivatives, reduced graphene oxides (rGOs). In addition, other two-dimensional nanomaterials such as carbon nitrogen (C_3_N_4_), boron nitride (BN), and graphdiyne (GDY) have also been reported in electrocatalytic NRR.

This paper provides a broad review of the application of highly efficient N_2_ reduction electrocatalysts based on 2D non-metallic nanomaterials. Firstly, we introduce the detailed process and mechanism of electrocatalytic devaluation of nitrogen to ammonia. We elaborate on the issues and details to which we need to pay attention to realize the efficient electrocatalytic ammonia synthesis under mild conditions. We summarize the current indicators for measuring electrocatalysts. In addition, we describe the performance and reactivity of electrocatalysts based on 2D non-metallic nanomaterials. Through theoretical simulation and experimental verification, we summarize the two aspects and the construction of high-efficiency NRE (nitrogen reduction efficiency) electrocatalysts based on 2D non-metallic nanomaterials. A series of examples cover the application of various 2D non-metallic nanomaterials and other materials in the modification of NRR. Finally, we also introduce the challenges and future development opportunities based on 2D non-metallic materials in the field of electrocatalytic nitrogen fixation.

## 2. The Process and Mechanism of Nitrogen Reduction Reaction

Generally, in nature, the nitrogen fixation of plants and other microorganisms is a process of fixing N_2_ by nitrogenase. The process of this reaction is shown in Formula (1). This process requires 8 electrons and 8 protons and 16 equivalents of adenosine triphosphate (ATP). It can be seen from this process that it is very difficult to convert one N_2_ to two NH_3_ [24].

(1) $$N_{2}+{\;8H}^{+}+{8e}^{-}+\;16\mathrm{ATP}\;\to {\;2\mathrm{NH}}_{3}+{\;H}_{2}+\;16\mathrm{ADP}\;+16\mathrm{Pi}$$

At present, the Haber–Bosch process of ammonia synthesis in the world is accomplished directly through the combination between the dissociation of N_2_ and H_2_. The process is shown in Equation (2) [7].

(2) $$N_{2}+{\;3H}_{2}\to {2\mathrm{NH}}_{3}$$

As shown in Equation (2), the energy and thermodynamic temperature required by the industrial synthesis technology are very high. The recently developed low-energy-based synthesis of ammonia by electrocatalytic nitrogen reduction is shown in (3).

(3) $${2N}_{2}+{\;6H}_{2}O\;\to {\;4\mathrm{NH}}_{3}+{\;3O}_{2}$$

**Figure 1:**
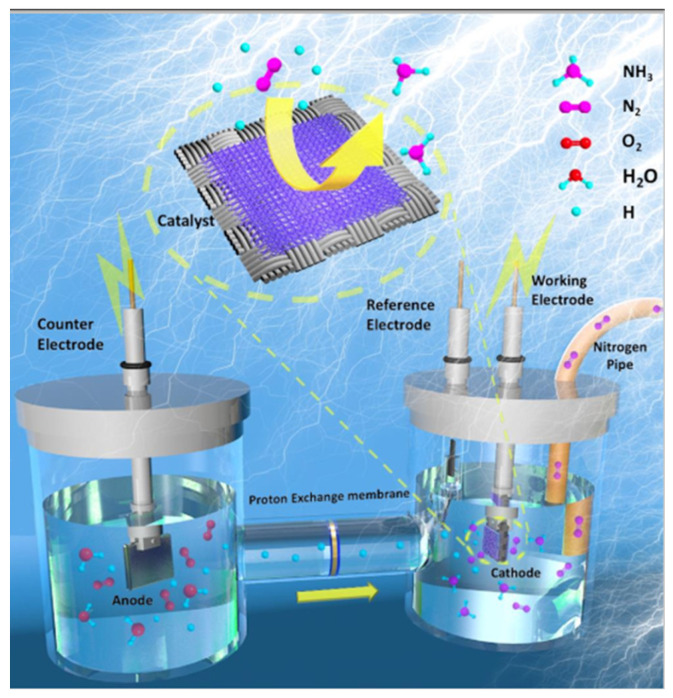
Schematic Diagram of NRR system [25].

Electrocatalysis is an auspicious approach that uses an electrical pulse to produce ammonia under calm conditions; these electrical pulses are generated by renewable resources. Generally, the NRR electrocatalytic approach adopts the method of nitrogen reduction on the cathode side. As shown in Equation (4), the entire process is carried out in an aqueous electrolyte. It requires 6 electrons and 6 protons to complete ammonia synthesis and water provides protons, and the reaction kinetics of the process is relatively slow. The reaction schematic is shown in Figure 1. Some recently reported catalysts are listed in Table 1.

(4) $$N_{2}+{\;6H}^{+}+{\;6e}^{-}\to {\;\mathrm{NH}}_{3}$$

**Figure 2:**
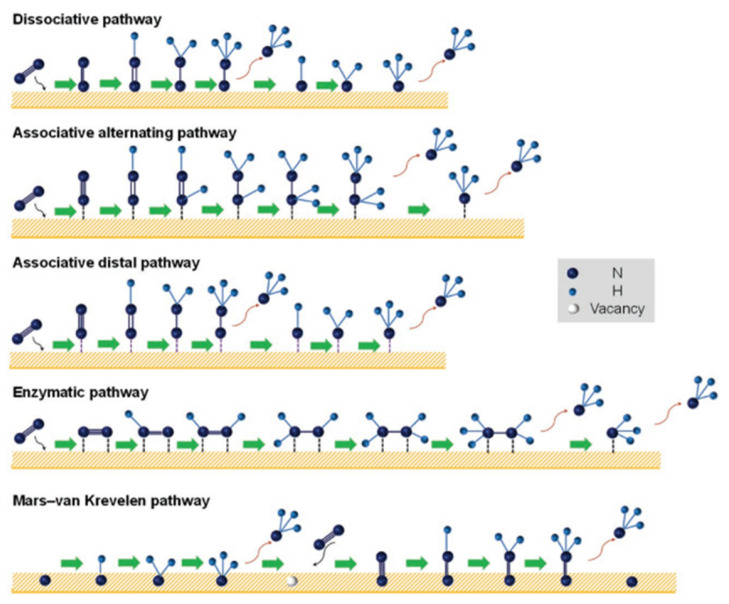
Electrocatalytic NRR reaction mechanism [26].

The mechanism of nitrogen reduction is generally recognized as follows: dissociative pathway, associative pathway, and Mars–van Krevelen pathway [26], as shown in Figure 2. After more than one hundred years of development, the dissociation mechanism explored is that of the current major ammonia synthesis industry, and the Haber–Bosch process follows this mechanism. In the dissociation mechanism, the breaking of high-energy bonds in nitrogen is a critical step. The broken single nitrogen molecules are left behind on the surface of the catalyst, and then hydrogenation is gradually performed to obtain NH_3_. The associative pathway is composed of the distal, alternating (single adsorption mode), and enzymatic (double adsorption mode) pathways. In other related approaches, two nitrogen atoms combine with each other, while in this approach nitrogen molecules stick and absorb on the surface of the catalyst and undergo hydrogenation. The associative approaches were divided into different pathways and these pathways will produce parallel pathways. In this approach, a nitrogen atom reacts with hydrogen atoms and produces two NH_3_ molecules from a single pathway simultaneously. In the enzymatic pathway, dinitrogen is coordinated side by side, followed by a hydrogenation step like the alternative pathway.

## 3. The Measurement Standards of Electrocatalyst Performance

There are three key activity indicators for measuring catalyst standards: stability, ammonia yield, and Faraday efficiency. Establishing a standard measurement system is especially important for studying electro-catalytic NRR. On the basis of this, the detection and measurement methods are summarized.

### 3.1. Stability

During the use of the catalyst, stability is one of the most important indicators. At present, all studied electrocatalysts are stable for approximately 24 h, which is still far from the requirements for electrocatalytic nitrogen infatuation to ammonia. The stability of catalyst during use determines the life of the electrocatalyst. When a voltage is applied to the electrocatalyst, the surface is poisoned by other non-effective components, which will cause the electrocatalyst to deactivate. Therefore, it is particularly important to design highly stable electrocatalysts. It can be considered that the electrocatalyst is not covered by other substances during continuous use and affects the active site of nitrogen adsorption. Understanding electrocatalysts is the way to obtaining essential information for designing synthetic electrocatalysis with high stability.

### 3.2. Faraday Efficiency

The electrocatalytic NRR Faraday efficiency attribute is the ratio of Faraday current used to synthesize ammonia in the total current. Because there is a very competitive reaction with HER in the synthesis of electrocatalytic synthesis of ammonia, when measuring the performance of electrocatalysis for the synthesis of ammonia, the conversion of NRR electrocatalyst is measured by calculating the Faraday efficiency of the process. This process is the reduction of nitrogen to NH_3_ by six-electron transfer. The Faraday efficiency can be calculated as follows (1):

(5) $${\mathrm{FE}}_{\mathrm{NH}3}\left(\%\right)=\frac{3\times F\times c\times V}{M_{\mathrm{NH}3}\times Q}$$

In Equation (5), F is the Faraday constant whose value is 96,485.30 C mol^−1^, C denotes ammonia concentration, M denotes molecular mass of ammonia, Q is total charge, and V is the electrolyte volume.

### 3.3. Ammonia Yield

The NH_3_ yield is a measure of the rate of electrocatalyst ammonia production and is an important indicator of its performance. Ammonia yield shows the NH_3_ yield per catalyst loading and unit time (unit electrode surface area). Table 1 shows the ammonia production rate of a series of electrocatalysts.

The formula for the ammonia production rate is as follows in Equation (4):

r_NH3_ = (n × V)/(t × m_cat_) (6)

In Equation (6), n represents the ammonia concentration in electrolyte, t shows electrolysis time, V represents electrolyte volume, and m_cat_ represents total mass of catalyst. In-membrane catalyst electrode surface area was used instead of m_cat_.

**Table 1:** Application of electrocatalysts based on 2D non-metallic nanomaterials.

Catalyst	Potential (vs. RHE)	Electrolyte	NH_3_ Production Rate	FE	Ref.
B-doped graphene	−0.50 V	0.050 M H_2_SO_4_	9.80 µg/h cm^2^	10.80%	[27]
O-doped graphene	−0.55 V	0.10 M HCl	21.30 µg/h mg	12.60% (−0.45 V)	[28]
S-doped graphene	−0.60 V	0.10 M HCl	27.30 µg/h mg	11.50% (−0.50 V)	[29]
S dots-rGO	−0.85 V	0.50 M LiClO_4_	28.5 µg/h mg	7.070%	[30]
FeOOH QDs-GS	−0.40 V	0.10 M LiClO_4_	27.30 µg/h mg	14.6%	[31]
CeO_2_-rGO	−0.70 V	0.10 M Na_2_SO_4_	16.99 µg/h mg	4.78%	[32]
P-doped graphene	−0.65 V	0.50 M LiClO_4_,	32.3 µg/h mg	20.82%	[33]
MoO_2_/RGO	−0.35 V	0.10 M Na_2_SO_4_	37.40 µg/h mg	6.6%	[34]
CuO/RGO	−0.75 V	0.10 M Na_2_SO_4_	1.80 × 10 mol/s cm^2^	3.9%	[35]
N–S co-doped graphene	−0.60 V	0.10 M HCl	7.70 µg/h mg	5.8%	[36]
Pd_0.2_Cu_0.8_/rGO	−0.20 V	0.10 M KOH	2.81 µg/h mg	-	[37]
ZnO/RGO	−0.65 V	0.10 M Na_2_SO_4_	17.70 µg/h mg	6.4%	[38]
Ag NPs-rGO	−0.70 V	0.10 M Na_2_SO_4_	18.8 µg/h mg	3.60%	[39]
PTCA-rGO	−0.50 V	0.10 M HCl	24.70 µg/h mg	6.9%	[40]
Fe_2_O_3_-rGO	−0.50 V	0.50 M LiClO_4_	22.1 µg/h mg	5.89% (−0.40 V vs. RHE)	[41]
Mn_3_O_4_-rGO	−0.85 V	0.10 M Na_2_SO_4_	17.40 µg/h mg	3.52%	[42]
Cr_2_O_3_-rGO	−0.70 V	0.10 M HCl	33.30 µg/h mg	7.33% (−0.60 V vs. RHE)	[43]
TA-rGO	−0.75 V	0.50 M LiClO_4_	17.020 µg/h mg	4.83%	[44]
DG-800	−0.40 V	0.01 M H_2_SO_4_	4.3 µg/h mg	8.5%	[45]
SnO_2_/rGO	−0.50 V	0.10 M Na_2_SO_4_	25.60 µg/h mg (5.1 μg/h cm^2^)	7.10%	[46]
CoS_2_/NS-G	−0.05 V	0.05 MH_2_SO_4_	25 µg/h mg (−0.2 V vs. RHE)	25.90%	[47]
NiO/G	−0.70 V	0.10 M Na_2_SO_4_	18.60 µg/h mg	7.8%	[48]
rGO/Fe@Fe_3_O_4_/CP	−0.30 V	0.20 M NaHCO_3_	1.30 × 10^−10^ mol cm^−2^ s^−1^	6.25%	[49]
BCN	−0.30 V	0.10 M HCl	7.7 µg/h mg	13.80%	[50]
B nanosheet	−0.80 V	0.10 M Na_2_ SO_4_	13.2 µg/h mg	4.04%	[51]
β-boron	−0.14 V	0.10 M HCl	3.1 µg/h mg	4.84%	[52]
BNS	−0.80 V	0.10 M Na_2_SO_4_	13.2 µg/h mg	4.04%	[53]
B_4_C nanosheet	−0.75 V	0.10 M HCl	26.5 µg/h mg	15.9%	[54]
B_4_C-BGQDs	−0.45 V	0.10 M HCl	28.60 µg/h mg	16.7% (−0.35 V vs. RHE)	[55]
O-CN	−0.60 V	0.10 M HCl	20.1 µg/h mg	4.9%	[56]
black P nanosheet	−0.70 V	0.010 M HCl	31.3 µg/h mg	5.06% (−0.60 V vs. RHE)	[57]
BN nanosheet	−0.75 V	0.10 M HCl	22.40 µg/h mg	4.7%	[58]
(1T-MoS_2_/g-C_3_N_4_)	−0.30 V	0.10 M HCl	29.9 µg/h mg	20.48%	[59]

## 4. Advanced Electrocatalyst Designed by 2D Non-Metal-Based Nanomaterials

The composite nano-electrocatalyst construction based on the 2D non-metallic nanomaterials has great advantages compared with other nanomaterials such as nanotubes, nanoporous material, nanowires, etc. It is easier to modify the compound that has more uniformly exposed lattice, and at the same time, more electrocatalytic active sites can be exposed in the case of certain catalysts [60]. Similarly, 2D non-metals have an ordered and simple molecular structure that makes it easier to nominate active sites with the help of experimental investigations and theoretical simulations. For electrocatalytic nitrogen reduction reaction, non-metallic electrodes such as carbon and graphite electrodes are important for research purposes. In the following section, three types of electrocatalysts are summarized: graphene and graphdiyne-based electrocatalyst, boron nitride (BN)-based electrocatalyst, and 2D non-metallic based electrocatalyst.

### 4.1. Graphene and Graphdiyne-Based Electrocatalyst

Since graphene was successfully peeled off in 2004 [16], layered 2D nanomaterials have attracted extensive attention from researchers, and have been the focus of materials development in the past decade. As is well known, ultra-thin 2D nanomaterials are single-layer or several-layer sheet-like structures. Its lateral dimension is much larger than the longitudinal dimension, and it is larger than 100 nm and even tens of microns. This 2D structure material has high surface area, excellent electrical conductivity, and good performance in light, electricity, heat, and other aspects. In recent years, it has been widely used in storage devices and energy conversion optoelectronics, catalysis, sensing, biotechnology, and other fields. Graphene, graphdiyne, and other composite nanomaterials, due to their different preparation methods, endow different physical and chemical characteristics. The composite materials constructed based on such non-metallic 2D nanomaterials have been greatly developed in the field of electrocatalysis. At the same time, composite nanomaterials and reduced graphene have attracted great attention in the development and utilization of catalysts for the synthesis of NH_4_.

(a) Theoretical and simulation-based progress

Recently, 2D graphene and graphdiyne based on high stability and excellent conductivity, have been investigated as the carrier of atomic catalysts [61,62,63]. Graphene nanomaterials doped with other atoms have been obtained as very good performance predictions for synthesis of ammonia in theoretical studies, and it is expected that NRR electrocatalysts with excellent performance will be developed. In addition, it is predicted that excellent NRR electrocatalysts can be obtained by combining N-doped graphene and graphdiyne with other transition metals and non-metal atoms [64].

**Figure 3:**
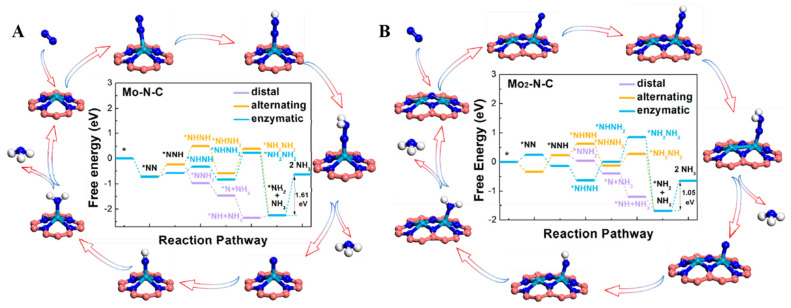
Free Energy representation for the nitrogen reduction reaction on the (**A**) Mo_2_-N-C and Mo-N-C (**B**) catalysts [61].

**Figure 4:**
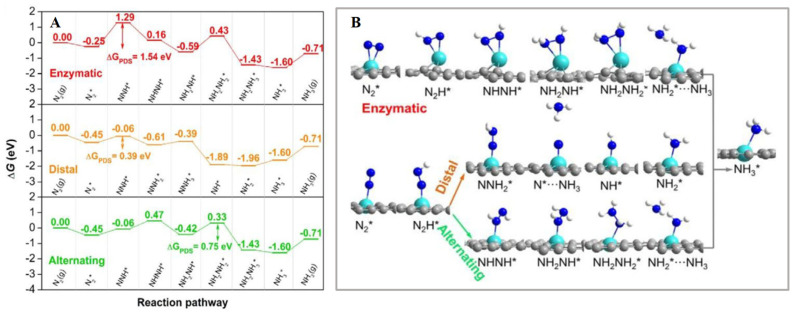
(**A**) The adsorption sites with the distal pathways on Nb-doped GD, shown by Enzymatic free energy diagram for nitrogen electrochemical reduction, Alternating, and Distal pathways on Nb@GD; (**B**) the ammonia conversion by nitrogen [62]. * represents an adsorption site.

Due to the high stability of N_2_ molecules, there is an essential demand to develop highly active electrocatalysts to reduce the energy consumption of NRR. He’s group [61] reported the use of binuclear atomic catalysts BAC on two-dimensional materials for electrochemical nitrogen reduction reaction. Using density functional theory, the authors demonstrated the adoption energies of nitrogen, NH_2_, and NNH obtained by N-doped graphene. Electrochemical NRR catalysts of different mononuclear atoms and binuclear atoms were carried out comparatively, and it was found that the Gibbs Faraday efficiency of NNH, N_2_, and ammonia on binuclear N-C and mononuclear catalysts were different. The results show that the Mo binuclear atomic catalyst is a good NRR electrocatalyst. However, due to the state of Mo atoms moving down relative to the Faraday efficiency, the difference in free energy on the three possible paths has confirmed that Mo BAC has a lower potential determination step than SAC (hydrogenation of NH_2_* to NH_3_), as shown in Figure 3A,B. Ma et al. [64], through first-principles calculations, systematically investigated the transition-metallic elements such as Fe, Co, Ni, and Mn monomers and dimers in graphdiyne monolayer for nitrogen reduction reaction electrocatalytic performance. As shown in theoretical results, stepped Ru (0001) surfaces have less catalytic activity than the transition metal monomer- and dimer-anchored monolayer. Infected cobalt-doped monolayer has the best electrocatalytic activity. During this procedure the initial potential is approximately −0.43 V, which is enough for HER reaction. Co_2_@GDY have good nitrogen removal reaction electrocatalytic activity by near state for Fermi level and electron donating ability. Similarly, these were studied by Han et al. [62] as electrocatalytic NRR conversion catalysts. After screening, the authors calculated that the free energy of the NRR surfaces of V@GD, Ti@GD, Nb@GD, W@GD and Ru@GD is relatively lower than that of the Ru surface. Among them, Nb-doped GD proved to be the suitable material for nitrogen reduction reaction, with a potential of 0.270 V, and despite a simple estimation of the HER competition by the Boltzmann distribution, it still has a high selectivity of 49.95%, proving the high selectivity of the product and the highly oriented product of NH_3_. The free energy spectra of Nb@GD and corresponding intermediates for electrochemical reduction of N_2_ in three ways are shown in Figure 4A,B. Moreover, Hui, L et. al investigated the graphdiyne-based electrocatalyst for the generation of ammonia [65,66,67,68,69]. Yuliang Li et al. demonstrated some significant guidelines for the design and development of GDY-based high-performance materials and devices in energy fields [70].

Zhen et al. [63] used first-principles calculations to show that a single heteroatom O-doped graphene (GDY) was used as the NRR electrocatalyst. The authors calculated, through DFT, that the oxygen injection in GDY resulted in a distribution of electron density (ED). The carbon molecules closest to the oxygen atoms increased the trapping ability of the N_2_ molecule. The results showed that the potential limiting steps do not change with strain. Within the strain range from −1% to 5%, the alternating mechanism is *NHNH_2_→*NH_2_NH_2_, and the remote mechanism is *NH_2_→*NH_3_. The limit potential is higher than the remote mechanism. The ultimate potential of the two mechanisms decreases with tensile strain.

(b) Experimental-based progress

Recently, Wang et al. [45] reported the application research of undoped defect graphene in N_2_ reduction. Through theoretical simulation and experimental verification, the defect site is the only one effective site for N absorption and activation. This shows that the defect site plays a crucial role in electrocatalytic activation of nitrogen atoms. It also proves that the construction of structural defects in 2D graphene is particularly important for NRR catalysts. The following subsections introduce graphene doped with other elements as well as 0D and 1D as excellent NRR electrocatalysts constructed by nanomaterials.

(i) Doping of B, S, P, N, and other non-metallic elements

Zheng et al. [27] proved that boron-doped graphene (BG) 2D nanomaterials are very good electrocatalytic nitrogen-fixing materials and can improve the absorption of nitrogen. The G skeleton composite with boron atoms can maintain the original sp^2^ hybrid and planar structure. The doping of element B results in electron defects in G, and the electron-deficient B site enhances the binding ability with N_2_ molecules, thereby improving electrocatalytic activity. The author revealed the boron-doped carbon structure electrically reduced nitrogen to the lowest energy barrier of ammonia. Boron-doped carbon structures have different concentrations. If the doping level is at 6.2% in boron-doped graphene, it will have a higher ammonia generation rate of approximately 9.80 μg/h cm^2^, with 0.50 V v and 10.8% Faraday efficiency level in aqueous level. Sun et al. [29] suggested S-doped graphene as an nitrogen reduction reaction electrocatalyst. In 0.11 molar hydrochloric acid, the catalyst obtains the high rate of ammonia yield of approximately 27.30 μg/h mg with scoring Faraday efficiency level of 11.51% (−0.5 V vs. RHE).

Sun et al. [33] published that the phosphorous-doped graphene also had the ability to produce the ammonia yield in 0.50 M solution of LiCIO_4_. The ammonia yield is approximately 32.33 μg/h mg, with the Faraday efficiency level of 20.82%, and potential is at −0.6 V. The PG provides electrons to enhance the electrocatalytic reduction of nitrogen. Wei et al. [36] published that nitrogen- and sulphur-doped graphene also act as a nitrogen reduction reaction electrocatalytic in which the ammonia yield is approximately 7.7 μg/h mg, with Faraday efficiency level of 5.8%. DFT and experiments verified that nitrogen–sulphur-doped composite creates more active sites, and its defects promote N_2_ adsorption and activation.

(ii) Recombination with 0D nanomaterials (quantum dots, clusters, and nanoparticles)

**Figure 5:**
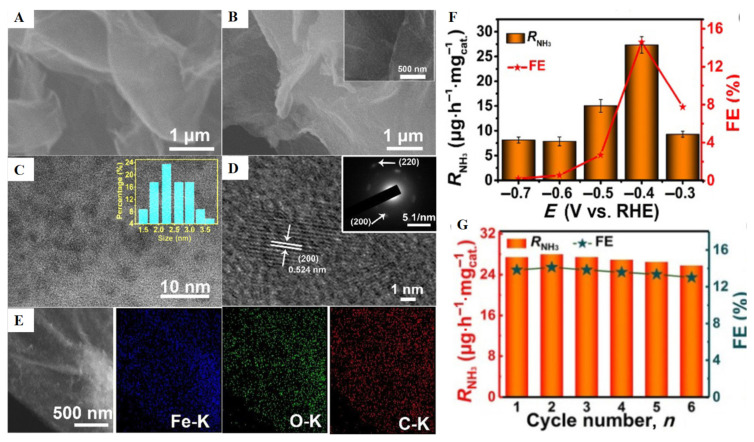
Scanning electron microscopy images of (**A**) GS and (**B**) FeOOH Quantum Dots-GS. (**C**) TEM image of FeOOH QDs-GS. (**D**) SAED and HRTEM images. (**E**) EDX elemental mapping & SEM images of carbon, iron, and oxygen in FeOOH Quantum Dots-GS. (**F**) Fes and RNH_3_ for FeOOH Quantum dots-GS/CP under different yields at −0.4 V using different electrode. (**G**) Recycle test of FeOOH Quantum dots-GS/CP at −0.40 volt [31].

According to recent reports, the nano catalysts supported on graphene can not only prevent the accumulation of nano catalysts, but also the synergy between the two components can improve the overall catalyst performance. Zhu et al. [31] synthesized the FeOOH quantum dots and then doped with graphene sheets, as shown in Figure 5A–E; these doped sheets have also good photocatalytic activity for NRR. During this process the NH_3_ yield is approximately 27.30 μg/h mg with the Faraday efficiency level of 14.6% in LiClO_4_ solution. As shown in Figure 5F, the catalyst shows the best persistence. Liu et al. [38] synthesized the zinc oxide quantum-dots-doped graphene oxide which also had good ammonia yield of approximately 17.70 μg/h mg with Faraday efficiency level of 6.4% in Na_2_SO_4_ solution, and voltage of approximately −0.64 V. In theoretical work it is highly expected that the composite of zinc oxide and reduced graphene have active and very stable catalysts for nitrogen reduction reactions. In experimental synthesis processes, many active sites are produced in ultra-fine composite of ZnO/rGO that are good for nitrogen absorption. DFT acknowledged that electronic coupling of reduced graphene and zinc oxide have great reduction in the energy barrier for stabilization of N_2_H. Chu et al. [46] investigated SnO_2_ quantum dots that were doped with reduced graphene and used them as nitrogen reduction reaction electrocatalysts. These quantum dots showed the poor hydrogen evaluation during the experimental procedure. The composite at −0.50 V in 0.10 Molar of sodium sulfate provided a NH_3_ yield of 25.6 μg/h mg and Faraday efficiency level of 7.1%. By the DFT computations, it was observed that tin oxide quantum dots with reduced graphene have some active sites for nitrogen atoms absorption. The electrical coupling of tin oxide QDs and reduced graphene increased conductivity while work function was reduced, thereby greatly abbreviating the energy boundary limits of *N_2_→*N_2_H, which is the rate determination path of the NRR procedure.

**Figure 6:**
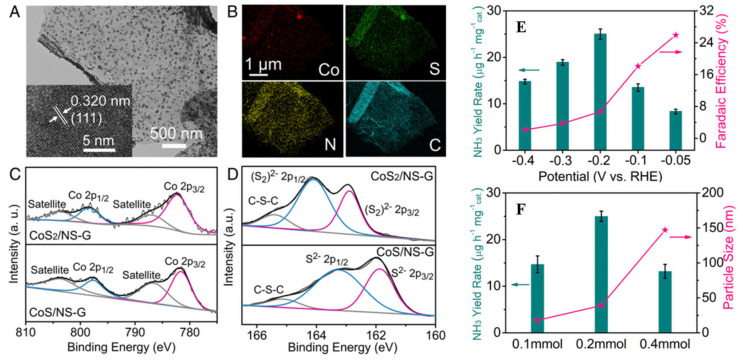
CoSx/NS-G hybrids composite characterization. (**A**) TEM and HRTEM images of CoS_2_/NS-G. (**B**) CoS_2_-doped NS-G hybrid composite elemental mapping images. XPS spectrum of Co 2p (**C**) andS 2p (**D**) for the CoS/NS-G and CoS_2_/NS-G hybrid products. (**E**) NH_3_ yield rate and Faradaic efficiency of CoS_2_/NS-G at each given potential. (**F**) Comparison of NH_3_ yield rate at −0.2 V and particle size for CoS_2_/NS-G hybrids which was synthesized by different amounts of cobalt salt. [47].

Yan et al. [37] synthesized the PdCu nano cluster doped on reduced graphene as a nitrogen reduction reaction electrocatalyst, in which palladium and Cu were added in different concentrations; from all the concentrations, Pd_0.2_Cu_0.8_/rGO had the best effects and the yield was approximately 2.80 μg/h mg at voltage of approximately −0.20 V vs. RHE. Mao et al. [32] synthesized the cerium nanoparticles and then deposited on the reduced graphene nanocomposite and used it as an electrocatalyst for nitrogen reduction reaction. The Faraday efficiency of CeO_2_-rGO produced the high yield of NH_4_, approximately 16.98 μg/h mg and voltage of approximately −0.70 and has excellent selectivity. At the same time, the research group combined silver nanoparticles with rGO (Ag NPs-rGO) [39] as a NRR electrocatalyst to maintain good stability and sustainability; rGO and its anchor dispersed on this carrier material (Ag NPs) enhances its conductivity. Therefore, introducing rGO into the catalyst can significantly improve the catalytic activity. In 0.10 Molar sodium sulfate solution, FE of Ag NPs-rGO is 3.6%, and the NH_3_ yield is 18.8 μg/h mg. In contrast, the FE of Ag NPs is 2.25%, and the yield of NH_3_ is 9.43 μg/h mg. Chu’s group developed a hybrid composite catalyst of molybdenum oxide NPs on rGO [34] and a composite catalyst loaded with CuO nanoparticles [35]. For MoO_2_/rGO in 0.15 Na_2_SO_4_ at 0.35 Volt, NH_3_ production was 37.40 μg/h mg, FE was 6.6%. The authors showed through DFT that, compared with MoO_2_ alone, it had a very high NRR activity. MoO_2_/rGO hybrids have a stronger electron interaction with *N_2_H and contribute negative charges from the active molybdenum site to *N_2_H, thereby greatly decreasing the energy boundary limits formed by *N_2_H, which is an identifying point in determining the nitrogen reduction reaction approach by potential energy. At the same time, the resulting CuO/rGO nanocomposites have excellent selectivity and high stability. At −0.74 Volt, CuO/rGO showed the impressive ammonia yield, which is approximately 1.80 × 10^−10^ mol/s^−1^ cm^2^ and the Faraday efficiency level was approximately 3.9%. Sun et al. [41] synthesized the nanocomposite of iron oxide and reduced graphene oxide for nitrogen reduction electrocatalyst; for this purpose LiClO_4_ solution was used and during this process a high amount of ammonia yield was produced, which is approximately 22.13 μg/h mg with Faraday efficiency level of 5.89% vs. RHE. On the basis of this, Wang’s group [49] synthesized the Fe@Fe_3_O_4_/carbonized paper with a layered reduced graphene oxide/eggshell structure and these were used as electrodes for electrocatalytic nitrogen reduction reaction in mild boundary limits. Electrocatalytic measurement results showed that the electrode had high electrocatalytic activity and the high amount of NH_3_ generation rate was approximately 1.30 × 10^−10^ mol/s^−1^ cm^2^, Faraday efficiency level of 6.25% vs. RHE, and better stability. The excellent electrocatalytic performance of this electrode for electrochemical fusion of NH_3_ is mainly characterized by its unique sandwich-like nanostructure, Fe@Fe_3_O_4_ nanoparticles with yolk–shell structure, and the synergy between rGO and Fe@Fe_3_O_4_. Wu et al. [47] successfully constructed a strong bridge bond (Co-N/SC) on the (NS-G), and proposed an interface engineering strategy. The design based on the special interface leads to faster reaction kinetics to achieve electrocatalytic nitrogen reduction reaction at low potential. Compared with the RHE at −0.05 Volt, CoS_2_-doped NS-G hybrid, as shown in Figure 6A–D, shows a high yield and FE to produce NH_3_. The highest FE is 25.9%, and NH_3_ production is 25 μg/h mg. As shown in Figure 6E,F, Chu et al. [48] synthesized the graphene-doped NiO nano dots that were used as electrocatalyst have good NH_3_ yield of approximately 18.60 μg/h mg and Faraday efficiency level of 7.8%. NiO nanoparticles have a main active center, but mono dots have more active sites.

(iii) Recombination with 1D nanomaterials (nanorods)

Reasonable design of effective electrocatalysts for NRR is particularly important. The hybridization strategy of 1D materials modified and doped with 2D graphene materials is essential to achieve efficient electrocatalytic NH_3_ synthesis. Recently, Luo et al. [40] proposed a PTCA-rGO nanohybrid composite used as a nonmetallic electrocatalyst. Due to the high electrochemical and structural stability electrocatalyst (PTCA-rGO) formed by the synergy of PTCA and rGO, at −0.5 Volt, in 0.10 Molar Hydrochloric acid, the PTCA-rGO hybrid can provide 24.70 μg/h mg. A large amount of ammonia has a FE of 6.90%. DFT estimates that NRR on the hybrid catalyst occurs through remote association and partial substitution.

### 4.2. Boron Nitride (BN)-Based Electrocatalyst

**Figure 7:**
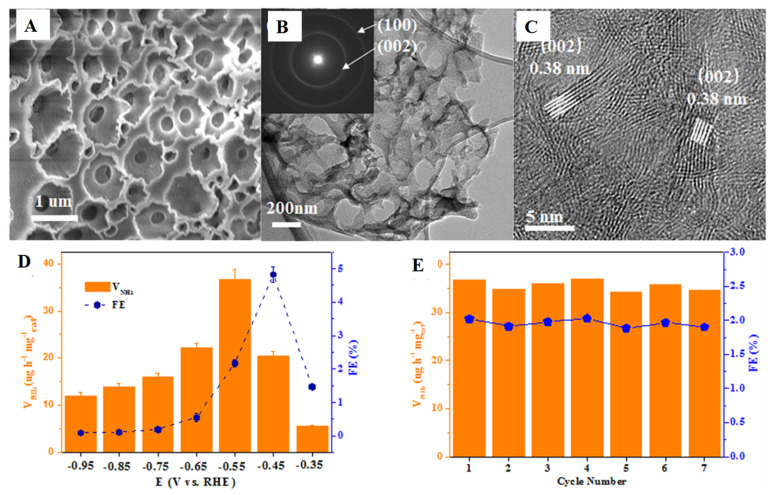
SEM, TEM, and HRTEM images of C-BN are shown in (**A**–**C**), respectively. (**D**) FEs and VNH_3_ and for C-BN/CP at given potential. (**E**) Recycle test of C-BN/CP at −0.5 Volt [76].

Boron nitride BN nanosheets and graphene have many essential advantages, such as oxidation resistance and chemical stability [71]. Boron nitride nanosheets have some defects such as N-vacancies and B-vacancies, which are also visible during electron beam irradiation [72,73]. Among them, the effect of B-vacancy formation is the best [74]. However, due to these point-like defects, BN nanosheets have good chemical activity that helps to support the metal particles with other catalytic reactions. Zhao et al. [75] theoretically studied the transition metals (Si, Ru, Mo, Pd, Rh, Ru, Ag, and Zn) loaded on boron nitride TM-BN through the DFT calculation. While investigating the possibility of vacant monolayers as N_2_ fixed electrocatalysts, the authors’ calculations showed that the single Mo atoms loaded on the defective BN nanosheets exhibit the highest N_2_ fixation catalytic activity under mild conditions by enzyme system, with an excessive potential of 0.19 Volt. Mo-embedded BN nanosheets resulted in high activity of the electrocatalyst on N_2_ fixation. Ma et al. [76] conducted extensive screening of NRR catalysts on defective hexagonal BN nanosheets loaded with a series of transition metal atoms through DFT calculations. Among them, the V/Tc atoms (V@BN and Tc@BN) doped on defective h-BN layer showed good NRR activity. The authors found that V-doped BN showed the better catalytic activity for NRR through the enzyme pathway, which was attributed to V-doping, and its overpotential was extremely low, only 0.25V. Liu et al. [77] synthesized the carbon-doped boron nitride nanosheets which had excellent electrocatalytic activity as shown in Figure 7A–C, as well as nitrogen durability fixation. In this process there is a high yield of ammonia generation of approximately 36.7 μg/h mg, and the voltage is approximately −0.55 V which indicates that the selectivity of the NRR is excellent (Figure 7D,E).

### 4.3. 2D Non-Metallic-Based Electrocatalyst

Recently, 2D single-element-based non-metallic nanomaterials, such as boron nanosheets, single-layer phosphorene, and black phosphorene (BP), due to their immense Faraday efficiency level and electronic structural representation, have been widely involved in the construction of NRR electrocatalysts. These 2D single-element non-metallic nanomaterials have been proposed by researchers as a promising active catalyst. The supported NRR transition metal monoatomic electrocatalyst provides theoretical guidance and experimental progress. Among them, the synergistic strategy with catalysts and other composite materials represents an effective way for the advancement of capable catalysts for electrochemical production of NH_3_. On the basis of the first principle DFT, Jiang et al. [78] predicted that the dimer clusters of Ti, Sc, and Fe supported on phosphorene are promising electrocatalysts for reducing N_2_ to NH_3_. In the electrochemical nitrogen reduction reactions applications, the material surface sites were reduced due to low intrinsic activity. Liu’s group [79] synthesized a single atom transition metal catalyst fixed on surface of BP, theoretically for NRR. By selecting one atom, tungsten doped with BP (W@BP) was found as a promising candidate for NRR. The mono-atomic W-doped BP has good activity on nitrogen reduction reaction. This remarkable performance was derived from the active site of WP_3_, which is responsible for an electron receiver and activates the nitrogen molecule by providing negative ions that enhance the charge transfer between BP and reaction intermediate.

Sun’s group [53] proposed boron nanosheets (BNS) with excellent selectivity and electrochemical durability as single-element 2D materials; when tested in 0.1 Molar Sodium Sulfate, at −0.80 Volt vs., boron nanosheets catalysts can achieve a Faraday efficiency level of 4.05% and have a high yield of ammonia approximately 13.22 μg/h mg. Sun’s group [52] reported that the 2D β-boron at different voltages have different yield but at 0.14 V relative to the RHE, boron nanosheets presented a maximal production of 3.1 μg/h mg. The ammonia Faraday efficiency level was 4.84%, which was greater than bulk boron. Wang’s group [50] confirmed, through experiments and theoretical calculations, that doped B-N provides sufficient active sites for carbon nanosheets, while at the same time preventing the competitive HER process. NRR with high ammonia generation rate of approximately 7.7 μg/h mg and FE of approximately 13.79% can be obtained at −0.30 Volt.

For nitrogen reduction reaction catalysts, non-metallic materials are the most favorable candidates due to their stability and efficient properties. Two-dimensional nano non-metallic materials are broadly used with some doping heteroatoms. These approaches opened a new gateway for the synthesis of electrocatalysts and two-dimensional materials. Wang’s group [80] investigated the boron-doped C_9_N_4_ monolayer films with the help of first-principles calculations [80]. However, boron-doped C_9_N_4_ have metallic properties and the ability to transfer charges during reduction because of their high photo activity, thermal conductivity, and stability. In other words, boron-doped C_9_N_4_ is a non-metal nitrogen reduction catalyst.

Sun’s group [54] synthesized the B4C nanosheets for the formation of ammonia with approximate yield of 26.5 μg/h mg and Faraday efficiency level of 19.56% vs. RHE. These nanosheets have high electrochemical activity. For this introduction reaction, Na_2_SO_4_ solution was used. This catalyst has the best selectively and activity. Xia’s group [55] studied in situ formation of B-GO BG with high selectivity and electrochemical stability on B_4_C nanosheets followed by cutting boron-doped graphene sheets into B-GO QDs. At the voltage of 0.44 Volt, in 0.10 M Hydrochloric acid under diffusive conditions, the ammonia yield was 28.60 μg/h mg, and Faraday efficiency was 16.7%. Sun’s group [56] synthesized the oxygen-doped carbon nanosheets for the purpose of nitrogen reduction reaction and achieved NH_3_ yield of 20.1 μg/h mg. Compared with RHE at −0.6 Volt in 0.1 M Hydrochloric acid under influence conditions, the FE was as high as 4.97%.

## 5. Conclusions and Outlook

The electrochemical method is a method for transforming high value-added products into green sustainable development. The electrocatalytic synthesis of ammonia developed in recent years takes advantage by reducing N_2_ to a high value-added product NH_3_. The introduction of this method has changed the traditional method of industrial synthesis of ammonia and is expected to be applied in the future. Faced with the challenge of highly efficient electrocatalysts, this manuscript is based on the advantages of 2D materials and the economic feasibility, environmental friendliness, non-corrosiveness, and unique physical and chemical properties of 2D non-metallic nanomaterials. A series of 2D non-metal-based nanomaterial electrocatalysts for nitrogen fixation are reviewed. Ideal electrocatalysis has an active site that efficiently adsorbs and activates N_2_ molecules. Increasing defects and exposed active sites are the key to selecting catalysts. This design strategy includes atomic doping on the surface of 2D non-metallic nanomaterials and synergy with doping substances. Similarly, the defect vacancy formed by the construction of the interface between the 2D material and the composite material is a potential catalytic activity center. At present, some materials with the selectivity of inhibiting HER to improve NRR have been developed, but there are still many incomplete structure–activity relationships for catalyst synthesis. The choice of nitrogen-fixing electrocatalyst and suppression of HER is still a huge challenge. There is an urgent need to develop some in situ characterization techniques to deeply analyze the structure–effect relationship of the nitrogen fixation process. For the low-yield, low-FE electrocatalysis process, there are many artifacts caused by interference results such as detection methods, which lead to a series of non-repeatable experimental phenomena. Electrocatalysis also has low stability and other shortcomings. In short, the future development of electrochemical synthesis of NH_3_ will depend largely on the establishment of a more efficient catalytic system, but it will remain a huge challenge for a long period.
